# Epigenetic silencing of *S100A2* in bladder and head and neck cancers

**DOI:** 10.18632/oncoscience.140

**Published:** 2015-03-16

**Authors:** Juna Lee, Piotr T. Wysocki, Ozlem Topaloglu, Leonel Maldonado, Mariana Brait, Shahnaz Begum, David Moon, Myoung Sook Kim, Joseph A. Califano, David Sidransky, Mohammad O. Hoque, Chulso Moon

**Affiliations:** ^1^ Graduate Program in Human Genetics and Molecular Biology, The Johns Hopkins University School of Medicine, Baltimore, MD, USA; ^2^ Department of Otolaryngology – Head and Neck Surgery, The Johns Hopkins University School of Medicine, Baltimore, MD, USA; ^3^ Department of Oncology and the Sidney Kimmel Comprehensive Cancer Center, The Johns Hopkins University School of Medicine, Baltimore, MD, USA; ^4^ Department of Urology, Johns Hopkins University, Baltimore, Maryland, USA; ^5^ Department of Pathology, Johns Hopkins University, Baltimore, Maryland, USA; ^6^ Milton J. Dance Head and Neck Center. Greater Baltimore Medical Center, Baltimore, Maryland, USA

**Keywords:** S100A2, methylation, head and neck cancer, bladder cancer, epigenetics

## Abstract

*S100A2*, a member of the *S100* protein family, is known to be downregulated in a number of human cancers, leading to its designation as a potential tumor suppressor gene. Here, we investigated the expression and methylation status of *S100A2* in head&neck and bladder cancer. Reduced mRNA and protein expression was observed in 8 head&neck and bladder cancer cell lines. To explore the mechanism responsible for the downregulation of *S100A2*, we treated six cell lines with 5-aza-2′-deoxycytidine. We found *S100A2* is silenced in association with aberrant promoter-region methylation and its expression is restored with 5-aza-2′-deoxycytidine treatment. Of 31 primary head&neck cancer cases and 31 bladder cancer cases, promoter methylation was detected in 90% and 80% of cases, respectively. Interestingly, only 1/9 of normal head&neck tissues and 2/6 of normal bladder tissues showed promoter methylation. *S100A2* promoter methylation can be detected in urine and is more frequent in bladder cancer patients than in healthy subjects (96% vs 48% respectively). Moreover, increased methylation of *S100A2* is linked to the progression of the tumor in bladder cancer (p<0.01). Together, this data shows that methylation-associated inactivation of *S100A2* is frequent and may be an important event in the tumorigenesis of head&neck and bladder cancer.

## INTRODUCTION

The S100 family is a large subgroup of Ca2+-binding proteins. There are at least 25 different members, displaying from 16% to 98% homology in sequence identity [1]. S100 proteins have been implicated in pleiotropic cellular events, with specific functions for each of the family members such as cell cycle regulation, cell growth, cell differentiation, and motility [1]. In addition, a range of different human diseases has been associated with changes in the expression of S100 genes, including cancer [2]. For example, *S100B* is known to be upregulated in melanomas and is used as a tumor marker [3]. Similarly, S100A4 is reported to be overexpressed in metastatic breast cancer cell lines and human tumor tissues [2]. Although many S100 genes are located in a cluster in chromosome 1q21 there is no evidence that their expression is synchronized by any manner. On the contrary, in a given cell type one *S100* can be highly expressed and other neighboring *S100* gene may be expressed at low level or unexpressed [4].

High and moderate expression level of S100A2 protein (also known as S100L or CaN19) was first observed in lung, kidney, liver, heart, and skeletal muscle with low levels detected in brain and intestine [5]. While *S100A2* is expressed in many normal tissues, its aberrant expression in a number of tumor tissues has been reported [3, 6].

The expression level of different S100 gene family members in cancer tissues seems to be regulated by epigenetic mechanisms. Among proteins which expression is effected by promoter methylation are *S100A4*, *S100A6*, *S100A10*, S100P which were shown to aberrantly hyper- or hypomethylated in breast cancer, colon, pancreas, prostate, gastric and endometrial cancer, among others [4]. *S100A2* appears to be gene particularly targeted by hypermetylation. In breast and lung cancers, treatment of cell lines with the demethylating agent 5-aza-2′-deoxycytidine led to the re-expression of *S100A2* mRNA, indicating that the lack of *S100A2* expression may be at least partially associated with aberrant methylation of the promoter region [7, 8, 9]. In addition, *S100A2* appears to positively regulate p53 transcriptional activity, while S100A4 expression strongly inversely correlates with p53 expression [10, 11]. Such evidence has led to the belief that *S100A2* may be candidate tumor suppressor gene.

While the methylation status of *S100A2* has been examined extensively in breast, lung, and prostate cancers, it has not been investigated in other cancers. In this study, we examined the status of 5′CpG island methylation of *S100A2* in head&neck and bladder cancer. We show that loss of *S100A2* expression in head and neck and bladder cancer cell lines is associated with the methylation of CpG islands and can be restored with 5-aza-2′-deoxycytidine treatment. Furthermore, we demonstrate high frequency of aberrant methylation of *S100A2* in primary head&neck and bladder tumor samples.

## RESULTS

### *S100A2* gene is silenced by hypermethylation in cancer cell lines

**Figure 1:**
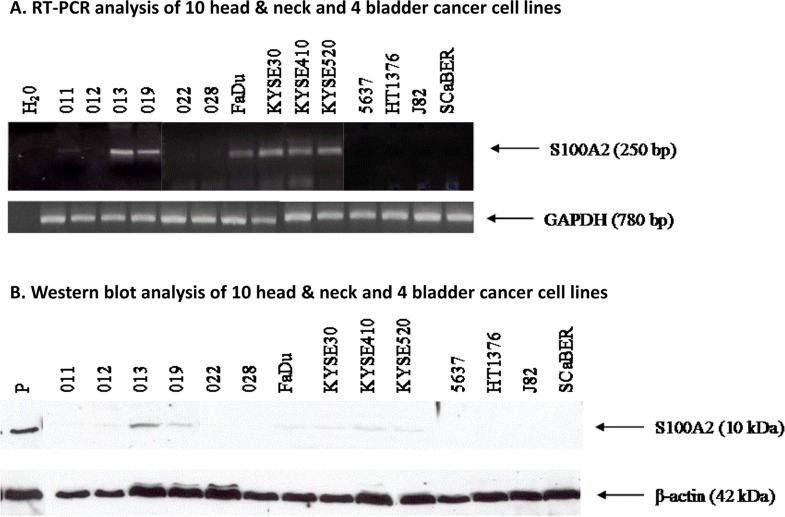
Expression of *S100A2* in head/neck and bladder cancer cell lines (A) RT-PCR was performed using sense and antisense primers for *S100A2* for 10 head/neck cancer cell lines (011, 012, 013, 019, 022, 028, Fadu, KYSE30, KYSE410, and KYSE520) and 4 bladder cancer cell lines (5637, HT1763, J82, and SCaBER). The name of each cell line is marked on the top of each lane, and the size of PCR product is marked with an arrow. PCR mixtures without templates were used as negative controls (H 0). GAPDH was the loading control. Each PCR product was cloned and its sequence confirmed. Eight of the 14 cell lines did not demonstrate expression of *S100A2*. (B) Western blot analysis was performed using antibodies for *S100A2* for the same 14 cancer cell lines. The name of each cell line is marked on the top of each lane, and the size of the protein is marked with an arrow. MDBK cells were used as positive control for Western blots (P). β-actin was the loading control.

Using RT-PCR and Western blotting, we analyzed the expression patterns of *S100A2* in 14 established head&neck and bladder cancer cell lines (Figure 1A and 1B). *S100A2* expression was absent in four head and neck cancer cell lines (011, 012, 022, and 028) and all four bladder cancer cell lines (5637, HT1376, J82, and SCaBER). It was expressed in the other six head and neck cancer cell lines (013, 019, Fadu, KYSE30, KYSE410, and KYSE520).

**Figure 2:**
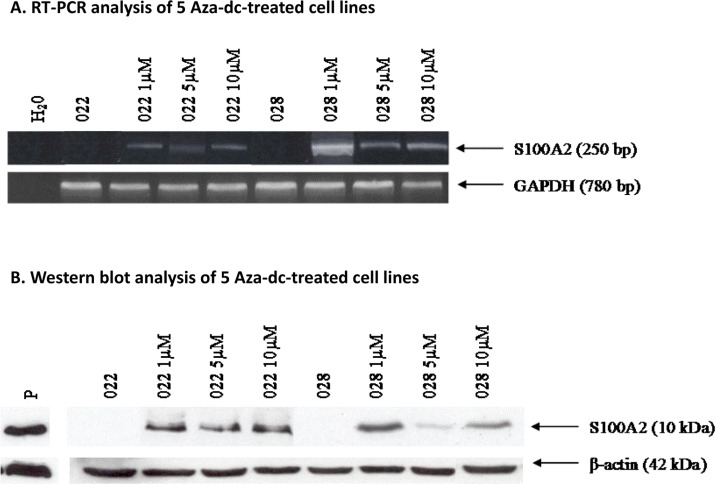
Expression of *S100A2* in head&neck cancer cell lines after treatment with 5-aza-2′-deoxycytidine The head&neck cancer cell lines 022 and 028 were treated with 1, 5, and 10 μM of 5′-aza-2′-deoxycytidine for 3 days. (A) RT-PCR was performed using sense and antisense primers for *S100A2*. The name of each cell line and the amount of 5′-aza-2′-deoxycytidine treatment is marked on the top of each lane. The size of PCR product is marked with an arrow. PCR mixtures without templates were used as negative controls (H 0). GAPDH was the loading control. Each PCR product was cloned and its sequence confirmed. Both cell lines demonstrated re-expression of *S100A2* after treatment with 5′-aza-2′-deoxycytidine. (B) Western blot analysis was performed using antibodies for *S100A2* for 022 and 028 after treatment with 5′-aza-2′-deoxycytidine. The name of each cell line and the amount of 5′-aza-2′-deoxycytidine treatment is marked on the top of each lane. The size of the protein is marked with an arrow. MDBK cells were used as positive control for Western blots (P). β-actin was the loading control.

Thereafter, we performed pharmacological unmasking to verify if treatment with DNA methylation inhibitor 5-aza-2′-deoxycytidine (5Aza-dC) can lead to *S100A2* re-expression. Six cell lines which did not express *S100A2* (022, 028, 5637, HT1376, J82, and SCaBER) were treated with 5Aza-dC. In all of the treated cell lines, treatment with 5Aza-dC resulted in a robust re-expression of *S100A2*. Representative results for the head&neck cancer cell lines 022 and 028 are shown in Figure 2.

Then, we investigated the *S100A2* promoter methylation status in all cell lines using bisulfite sequencing. Hypermethylation of the *S100A2* promoter was found in the eight cancer cell lines which were previously found not to express *S100A2* (011, 012, 022, 028, 5637, HT1376, J82, and SCaBER). As expected, no hypermethylation was detected in the cell lines that expressed *S100A2*. Quantitative methylation-specific PCR (QMSP) was then performed to determine more specifically the degree of promoter region methylation. The QMSP results were consistent with our bisulfite sequencing results; higher methylation values were detected in 8 cell lines (011, 012, 022, 028, 5637, HT1376, J82, and SCaBER), all of which had no *S100A2* expression in RT-PCR or Western blotting analysis, whereas the head&neck cancer cell lines 013, 019, Fadu, KYSE30, KYSE410, and KYSE520, which express *S100A2*, were unmethylated in this promoter region. Incidence of promoter hypermethlation as observed in QMSP is summarized in Table 1.

**Table 1:** Summary of *S100A*2 5′-CpG island hypermethylation in cell lines, primary tumors, and normal tissues

Tissue	Cancer cell lines (%)	Quantitative MSP	
		Tumor (%)	Normal (%)
head and neck	6/10 (60)	28/31 (90)	1/9 (11)
bladder	2/4 (50)	25/31 (80)	2/6 (33)
Grade I of bladder cancer	NA	1/3 (33)	NA
Grade II	NA	1/3 (33)	NA
Grade III	NA	11/13 (85)	NA

### *S100A2* hypermethylation in primary head&neck and bladder tumor tissue samples

To determine whether *S100A2* promoter methylation was limited to cultured head&neck and bladder cancer cell lines, we examined the promoter methylation of *S100A2* in primary head&neck and bladder cancers. Using QMSP, we examined the methylation status of *S100A2* in 31 head and neck and 31 bladder tumor tissue samples as well as in 15 normal tissue samples from the head and neck and bladder. The frequency of methylation was significantly higher in the primary tumor samples as compared to the normal tissue samples (Table 1). 90% (28 of 31) of primary tumor samples from the head and neck demonstrated methylation while 11% (1 of 9) of normal head and neck tissues were methylated (p<0.01). In the bladder, 80% (25 of 31) of the primary tumor samples showed methylation while 33% (2 of 6) of the normal bladder tissues were methylated (p=0.017).

We correlated DNA hypermethylation of *S100A2* in cancer samples with clinical and histopathological variables to determine whether these alterations were associated with any particular phenotype. In bladder cancer, the frequency of methylation was found to be significantly higher as the tumor grade increased (p<0.01). In the primary bladder cancer samples, methylation occurred in 33% of grade I (1 of 3) and grade II (1 of 3) urothelial cell carcinomas, whereas methylation occurred in 85% (11 of 13) grade III carcinomas. These data suggest that the loss of *S100A2* expression may be an important event during the cancer progression. Aberrant *S100A2* methylation in primary head&neck and bladder tumors had no correlation with patient demographic data, including age and gender, histological subtype, and staging of the tumor (data not shown).

### *S100A2* methylation in DNA isolated from urine in bladder cancer patients

To investigate whether *S100A2* methylation could be potentially utilized for detection of bladder cancer, we investigated *S100A2* methylation status in DNA isolated from urine samples. High frequency of methylation was observed in urine samples from cases in comparison with controls (96%vs. 48%; p<0.01, Chi-square test) (data not shown).

## DISCUSSION

We have found aberrant methylation involving the promoter regions of *S100A2* in different histologies of head&neck and bladder cancer. In cancer cell lines, DNA promoter hypermethylation correlated with loss of gene expression and could be efficiently restored with the de-methylation agent, 5-aza-2′-deoxycytidine. In addition, our findings of significantly high frequency of *S100A2* methylation in primary head&neck and bladder cancer support the fact that epigenetic silencing of this gene may be a tumor-specific event. This result fits the paradigm that has now been widely documented in many malignancies of a reciprocal relationship between the density of methylated cytosine residues in the 5′ region of some gene promoters and the transcriptional activity of that gene. We also found *S100A2* methylatation cases in normal head&neck and bladder tissue in 1 of 9 head&neck and 2 of 6 normal bladder tissues. At present we have no clear explanation of *S100A2* promoter methylation in cases of normal head&neck and bladder tissue. Environmental factors or aging process may be responsible for this kind of alterations which may be a very early stage of cellular transformation. It should be noted that *S100A2* promoter hypermethylation has previously been demonstrated in prostate cancer, but considerable levels of methylation were also present in some non-malignant prostate tissues [12].

Expression profiles of *S100A2* indicate that it may be a candidate tumor suppressor gene [12, 13]. The role of *S100A2* as a tumor suppressor is supported by study of *BRCA1* mutant and basal-like breast cancer cell lines, in which *S100A2* exogenous expression resulted in growth inhibition, while siRNA knockdown enhanced proliferation [14]. *S100A2* transfection in melanoma cells revealed its anti-proliferative activity [15], in gastric cancer cells it seems to decrease cell invasiveness [16], and similarly in head and neck squamous cell carcinoma *S100A2* reduces proliferation, cell motility and cancer cell invasion [17].

Additionally, aberrant *S100A2* expression in cancer has been demonstrated in many studies. In support of antionconcogenic role of *S100A2*, the expression level of this gene shown to be down-regulated in breast cancer, melanoma, prostate cancer, non-small cell lung cancer, esophageal squamous cell carcinoma and gastric carcinoma [3, 7, 8, 12, 16, 18]. However studies are not consistent, as other reports show considerable levels of *S100A2* mRNA and/or protein in non-small cell lung cancer [19], esophageal squamous cell carcinoma and Barrett's adenocarcinoma [20, 21], and gastric cancer [22]. Moreover, other studies identified *S100A2* up-regulation in a number of other malignancies, such as pancreas adenocarcinoma [23] and ovarian cancer [24], among others. Data from these studies put putative *S100A2* antitumor role in question. It seems that its anti-proliferative role as a tumor suppressor may depend on particular cell and biological context and it needs to be further elucidated.

Potentially important role of *S100A2* in carcinogenesis has been suggested. While few studies describing genetic alterations in *S100A2* gene in cancer exist [25], epigenetic mechanisms could also account for disturbance of *S100A2* function. In support of this, *S100A2* transcription repression in breast, lung, and prostate cancer cells and cell lines was shown to be mediated at least in part by site-specific methylation in the promoter region, similarly to a number of known and putative tumor suppressor genes [7, 9]. This report furthers these previous studies by demonstrating promoter region hypermethylation in head and neck and bladder cancer cell lines and primary cancer tissues. Our data show that *S100A2* expression is silenced in many cancer cell lines and can be restored by 5-Aza-dC treatment.

Interestingly, in prostate cancer the frequency of methylation was significantly higher in grade III tumors as compared to grade I and II tumors. This finding confirmed two previous reports on prostate adenomacarcinoma showing that loss of *S100A2* expression correlated with increasing tumor grade, suggesting that the loss of expression may be an important event in tumor progression [26]. Similarly, gradual loss of *S100A2* expression was found from gastritis, through intestinal metaplasia and dysplasia to gastric cancer [27]. In head and neck squamous cell carcinoma, *S100A2* expression was noted in some primary tumors, lymph node metastasis showed reduction in staining [28]. One the other hand, contrary to the results on differential *S100A2* expression in different grades of prostate adenocarcinoma, no difference in methylation status of *S100A2* was found between high-grade prostate intraepithelial neoplasia and benign prostate hyperplasia, in all of which observed methylation levels were consistently high [12]. This may suggest that not only methylation status is responsible for differential *S100A2* expression in different stages of prostate malignancy.

While our report provides a thorough examination of the expression of *S100A2* in head&neck and bladder, it should be noted that previous reports on the expression of *S100A2* in head&neck cancer have been contradictory. Studies found that the expression of *S100A2* was decreased in early-stage oral cancer cells and recurrent nasopharyngeal cancer [29, 30], however other investigators suggest *S100A2* upregulation in HNSCC [31, 32]. Studies of head and neck squamous cell carcinoma show variable levels of S100A2 in different cell lines [33]. Another study found this gene to be expressed in more than 95% of low-grade tumors and 51% of high-grade tumors of laryngeal squamous cell carcinoma, however *S100A2* negative cases were typically anaplastic non-keratinizing tumors that generally bear more malignant characteristics [34]. A study involving 424 normal and tumor tissues of various origin showed that while in normal non-epithelial tissues have low level of *S100A2* expression, in normal epithelial tissue its expression is present but decreases in tumors of epithelial origin concurrently with loss of keratin K14. This loss was more pronounced in glandular than squamous epithelial tissues [35]. This is consistent with data showing that *S100A2* is strongly expressed in epithelial basal cells and epithelial tumors of the skin [6, 36], and it was suggested that *S100A2* may play role in differentiation towards keratinocyte phenotype [37]. Furthermore, in a previous report, *S100A2* overexpression in squamous and basal cell carcinoma was often found to be associated with hyperplastic peri-lesional skin, and *S100A2* presence in this cases may reflect regenerative squamous differentiation [38]. Therefore, it seems that loss of *S100A2* might be an important event in tumor progression towards more malignant cell phenotype in head and neck cancer, but the decrease in its level may be also attributed to loss of epithelial phenotype.

As it was shown by many, tumor derived genetic disturbances can be detected in body fluids and used as cancer markers. Here, we investigated if hypermethylation of *S100A2* can be detected in urine samples of bladder cancer cases. Results presented here showed that frequency of hypermethylation of *S100A2* is significantly higher in urine samples from bladder tumor cases than in healthy subjects (96% vs 48% respectively). This may suggest that *S100A2* promoter methylation is a common event in bladder cancer and it can be detected in urine. However, due to the limited number of urine samples analyzed, we were not able to determine empirical cut off value to determine optimal sensitivity and specificity. Further study using large cohort of samples is needed to determine the feasibility *S100A2* promoter methylation as a screening marker in urine. Furthermore, analytical sensitivity needs to be determined before testing extended urine samples. In a recent study, while noting presence of *S100A2* methylation in head and neck squamous cell cancer tissue, very limited hypermethylation of *S100A2* was detected in saliva and serum in this malignancy [39]. Therefore, application of *S100A2* methylation for detection of head&neck and bladder cancers requires further evaluation.

In summary, we have demonstrated aberrant *S100A2* gene methylation in human head&neck and bladder cancer and most importantly promoter methylation of *S100A2* is inversely associated with gene expression. Because *S100A2* gene methylation is significantly more frequent in higher grade tumors, it may have a use as a marker of tumor progression.

## MATERIALS AND METHODS

### Cell lines, primary tumor and urine samples

The head and neck tumor cell lines Fadu, and bladder cancer cell lines 5637, SCaBER, HT1376, and J82 were obtained from ATCC (Manassas, VA). The head and neck cancer cell lines 011, 012, 013, 019, 022, and 028 were established in the Department of Otolaryngology – Head and Neck Surgery at The Johns Hopkins University. Head&neck cancer cell lines KYSE30, KYSE410, and KYSE520 were kindly provided by Dr. Shimada in the Department of Surgery, Kyoto University. 31 head and neck primary tumors and 31 bladder primary tumors from the Johns Hopkins Hospital were selected to determine the *S100A2* methylation frequency in primary tumors. Samples were stored frozen in −80 °C. 50 ml of voided urine were collected from 23 cases prior to definitive surgery at Johns Hopkins University School of Medicine and from 24 control patients with no history of genitourinary malignancy. Urine samples were centrifuged at 3000 X g for 10 minutes and the pallet was washed twice with phosphate-buffered saline (PBS) and frozen at −80 °C. Approval for the study was obtained from JHU Institutional Review Board.

### Bisulfite treatment and QMSP

Genomic DNA was isolated from cell lines, tissues and urine samples by digestion with proteinase K (0.5 mg/ml) in 1% SDS, Tris (1M, pH 8.8), EDTA (0.5M, pH 8.0), and NaCl (5M) overnight at 48°C followed by phenol/chloroform extraction and ethanol precipitation. Sodium bisulfite conversion of unmethylated cytosine residues to uracil of genomic DNA was performed. 2 μg of genomic DNA in 20 μl of H O containing 5 μg of salmon sperm DNA were denatured by incubation with 0.3M NaOH at 50°C for 20 min. DNA was then incubated at 70°C for 3 h in a 500 μl reaction mixture containing 2.5M sodium metabisulfite and 0.125M hydroquinone (pH 5.0). Treated DNA was purified with the Wizard DNA purification system according to the manufacturer instructions (Promega Corp., Madison, WI), and finally the bisulfite-modified DNA was resuspended in LoTE (2.5mM EDTA, 10mM Tris-HCL).

Bisulfite-modified DNA was used as a template for fluorescence-based real-time PCR (Taqman) as previously described [11]. Briefly, primers and probes were designed to specifically amplify the bisulfite-converted promoter of the gene of interest. The primer and probe sequences used for detection of target gene (*S100A2*, GenBank Accession #Y07755) and internal reference gene (*β-actin, ACTB*) are provided in Supplement Table 1.

The ratio of QPCR values of the gene of interest to β-*actin* was used as a measure of relative methylation level (target gene/*β-actin* × 1000). QPCR was performed in 20 μl reaction volumes containing 600nM of each primer; 200nM of probe; 0.75 units of platinum Taq polymerase (Invitrogen); 200μM of dATP, dCTP, dGTP, and dTTP each; 16.6mM ammonium sulfate; 67mM Trizma; 6.7mM MgCl_2_ 10mM mercaptoethanol; and 0.1% DMSO. 3 μl of treated DNA solution were used in each reaction and amplifications were carried out in a 7900 Sequence detector (Perkin-Elmer Applied Biosystems). PCR plates consisted of patient samples and multiple water blanks, as well as positive and negative controls. Leukocytes from a healthy individual were methylated *in vitro* with excess SssI methyltransferase (New England Biolabs Inc., Beverly, MA) to generate completely methylated DNA and serial dilutions of this DNA were used to construct calibration curves.

### Bisulfite sequencing

Bisulfite-treated DNA was subjected to PCR with primers flanking the targeted methylation-specific PCR regions. PCR products were then gel-purified and sequenced. Sequences of primers used for the amplification and sequencing are provided in Supplement Table 2.

### 5′-aza-2′-deoxycytidine treatment

022, 028, 5637, SCaBER, HT1376, and J82 were split to low density 48 h before treatment in 6-well plates. Cells were then treated for 3 days with 1, 5, and 10μM of 5′-aza-2′-deoxycytidine (Sigma) or were mock-treated with same volumes of 1X PBS. After the treatment, proteins and RNA were harvested.

### RNA isolation and RT-PCR

Total RNA was isolated using Qiazol reagent (Qiagen). Agarose gel electrophoresis at 1% and spectrophotometric analysis were used to assess RNA quality. First-stand cDNA was synthesized using random primers and M-MLV reverse transcriptase according to manufacturer's protocol with little modification (Invitrogen). cDNA was subjected to PCR using primers spanning exons 2 and 3 of *S100A2* (GenBank Accession number Y07755). Sequences of primers used for RT-PCR are provided in Supplement Table 3. As an internal control, Glyceraldehyde-3-phosphate dehydrogenase (GAPDH) was amplified to ensure cDNA quality and quantity for each RT-PCR. Final PCR products were resolved on a 2% TBE agarose gel and visualized.

### Western blot analysis

PBS-washed cells were lysed by sonication in ice-cold RIPA buffer (50mM Tris-HCl, pH 7.4, 150mM NaCl, 1mM PMSF, 1mM EDTA, 1% Triton X-100, 1% sodium deoxycholate, and 0.1% SDS). Protein concentrations were determined by the Bio-Rad DC protein assay (Bio-Rad). Equal amounts of protein were subjected to 16% SDS-polyacrylamide gel electrophoresis, and the proteins were transferred onto PVDF membranes. After blotting with 5% nonfat dry milk and 0.1% Tween-20 in 1X PBS, membranes were probed with mouse anti-*S100*L antibody (BD Transduction Laboratories) at a 1:1000 dilution in the blotting buffer for one hour at room temperature. After washing with 1X PBS and 0.1% Tween-20, the membranes were incubated with secondary antibody, HRP-conjugated anti-mouse antibody (Amersham), at a 1:5000 dilution in the blotting buffer. The membranes were again washed with 1X PBS and 0.1% Tween-20 and chemiluminescence system (Amersham) was used for protein detection. MDBK cell lysate provided by BD Transduction Laboratories was used as a positive control.

### Statistical analysis

Statistical tests were performed with Chi-square test using Statistica 10 software (Statsoft).

## SUPPLEMENTARY MATERIAL TABLES


