# Supplementary material for: Epigenetic silencing of S100A2 in bladder and head and neck cancers
Source: Oncoscience. 2015 Mar 16;2(4):410–8. doi: 10.18632/oncoscience.140 (PMC4468326; doi:10.18632/oncoscience.140)
Supplement: Supplementary file 1 [file oncoscience-02-0410-s001.pdf]

Epigenetic silencing of S100A2 in bladder and head and neck cancers

Supplement Table 1: Sequences of *S100A2* and *ACTB* primers and probes used for QMSP analysis.

	Sequence
S100A2 forward primer	5’-TGGTTTCGATTTTTTGATTTCG–3’
S100A2 reverse primer	5’–TCAAAATTCTTTTTACAACAACGCC–3’
S100A2 detection probe	6-carboxyfluorescein-5’–TCAAAATTCTTTTTACAACAACGCC–3’-6-carboxytetramethylrhodamine
ACTB forward primer	5’–TGGTGATGGAGGAGGTTTAGTAAGT–3’
ACTB reverse primer	5’–AACCAATAAAACCTACTCCTCCCTTAA–3’
ACTB detection probe	6-carboxyfluorescein-5’–ACCACCACCCAACACACAATAACAAACACA–3’-6-carboxytetramethylrhodamine

Supplement Table 2: Sequences of *S100A2* primers used for bisulfite converted-S100A2 amplification and bisulfite sequencing.

	Sequence
S100A2 forward primer	5’ – TGTTGGGATTATAGGAGTAAGTTAT – 3’
S100A2 reverse primer	5’ – ATCTCAAAATTCTTTTTACAACAAC – 3’
S100A2 sequencing primer	5’ – CCTAAACTAAAATATCCAAAAAAAA – 3’

Supplement Table 3: Sequence of *S100A2* primers used for RT-PCR.

	Sequence	Location relative to S100A2 transcription start site
S100A2 RT-PCR forward primer	5’ – CACTACCTTCCACAAGTACT – 3’	+41
S100A2 RT-PCR reverse primer	5’ – GAAGTCATTGCACATGACAG – 3’	+247
